# Book Reviews

**Published:** 1899-07

**Authors:** 


### *BOOK REVIEWS, PAMPHLETS, EXCHANGES.*

### *BOOK REVIEWS.*

[Contributions solicited for review.]

A TEXT-BOOK OF ANATOMY. By American authors. Edited by Frederic Henry Gerrish, M.D., Professor of Anatomy in the Medical School of Maine, Bowdoin College. Illustrated with 950 engravings in black and colors. Published by Lea Brothers & Co., Philadelphia.

We feel no hesitancy in saying that this is one of the best text-books on anatomy that has been issued for a long time.

The authors include the following well-known anatomists:

Arthur Dean Bevan, M.D., of Rush Medical College; Frederic Henry Gerrish, A.M., M.D., of the Maine Medical School; William Kuller, F.R.C.S. (Edin.), of the University of Texas; James Playfair McMurrich, A.M., Ph.D., of University of Michigan; George David Stewart, M.D., of Bellevue Hospital Medical College; George Woolsey, A.B., M.D., of Cornell University Medical College.

Most of the work has been done by Dr. Gerrish, and a large number of original illustrations are the products of his pen. Illustrations go largely to the making popular any work on anatomy, and it has been largely due to this one fact that the marked popularity of Gray's Anatomy has stood for so long a time. The illustrations in this work are excellent in the extreme, and with few exceptions have been taken from Testut's *Traité de l' Anatomie.*

We do not know when we have ever seen such excellent illustrations. The arteries are colored red and the veins blue, this arrangement being seen with every cut, and most of the illustrations show these colored plates. The book has been gotten up in an exceedingly attractive binding, and we predict for it a well-deserved popularity among students and teachers of anatomy.

ATLAS OF THE EXTERNAL DISEASES OF THE EYE, INCLUDING A BRIEF TREATISE ON THE PATHOLOGY AND TREATMENT. By Dr. O. H. Haab, of Zurich, Switzerland. Published by W. B. Saunders, Philadelphia. Price $3.00.

The name Haab is too well known to need any introduction, if for no other reason than being the originator of the powerful electro-magnet for removing particles of iron and steel from the eye. We have already as a valuable addition to our library Professor Haab's "Atlas of Ophthalmoscopy and Ophthalmoscopic Diagnosis," and now that this new atlas of external diseases has been published, the two together go hand in hand. In this last atlas the diseases common to the lids, lachrymal apparatus, conjunctiva, sclera, iris, cornea, tumors, etc., are pictured, and a brief description, with treatment, is given to each pathological condition. The colored plates representing each disease are works of art, and rarely have we ever seen truer pictures of inflammatory states just as they really are. Professor Haab remarks in his preface: "I fully appreciate how difficult it is to give a faithful reproduction of the external diseases of the eye. But after seeing the work of Mr. J. Fink, of Munich, at my clinic last summer, I became convinced of his ability to accomplish anything within the range of the illustrators' art." He was certainly not mistaken; the work is valuable to every physician.

---

AN EPITOME OF THE HISTORY OF MEDICINE. By Roswell Park, A.M., M.D., Professor of Surgery in the Medical Department of the University of Buffalo, etc. Based upon a course of lectures delivered in the University of Buffalo. Second Edition. Illustrated with Portraits and other Engravings. $6\frac{1}{2} \times 9\frac{1}{2}$ inches. Pages xiv.—370. Extra Cloth, $2.00 net. The F. A. Davis Co., Publishers, 1914-16 Cherry St., Philadelphia.

This noteworthy production of the History of Medicine is before us again in the second edition. To the student of medicine this is one of the most valuable works that has appeared within recent years. It should be in the library of every physician, and not only should it be there, but it should be read and studied thoroughly so that there should be no occasion for a lack of historical knowledge concerning the history of the healing art. The medical profession is under lasting obligations to Dr. Park for giving to them in such an entertaining form this history of medicine. No truer words can be found than those in the opening lines of the author's preface: "The history of medicine has been sadly neglected in our medical schools. The valuable and fruitful lessons which it tells of *what not to* do have been completely disregarded, and in consequence the same gross errors have over and over been repeated."

